# The Correlation of Serum Calpain 1 Activity and Concentrations of Interleukin 33 in COVID-19 Acute Respiratory Distress Syndrome

**DOI:** 10.3390/biomedicines11071847

**Published:** 2023-06-27

**Authors:** Domagoj Loinjak, Damir Mihić, Robert Smolić, Lana Maričić, Ines Šahinović, Martina Smolić, Renata Sikora, Sanja Loinjak, Kristijan Dinjar, Aleksandar Včev

**Affiliations:** 1 Faculty of Medicine, University J. J. Strossmayer in Osijek, 31000 Osijek, Croatia; loinjak.domagoj@gmail.com (D.L.); mihic27@gmail.com (D.M.); isahinovic11@gmail.com (I.Š.);; 2 Department of Pulmology and Intensive Care Medicine, University Hospital Centre Osijek, 31000 Osijek, Croatia; 3 Faculty of Dental Medicine and Health Osijek, University J. J. Strossmayer in Osijek, 31000 Osijek, Croatiamartina.smolic@mefos.hr (M.S.); renatasmolic0@gmail.com (R.S.); sanja.loinjak@gmail.com (S.L.); aleksandar.vcev@fdmz.hr (A.V.); 4 Department of Heart and Vascular Diseases, University Hospital Centre Osijek, 31000 Osijek, Croatia; 5 Department of Clinical Laboratory Diagnostics, University Hospital Centre Osijek, 31000 Osijek, Croatia; 6 Department of Maxillofacial and Oral Surgery, University Hospital Centre Osijek, 31000 Osijek, Croatia

**Keywords:** ARDS, COVID-19, interleukin 33, calpain 1

## Abstract

Acute respiratory distress syndrome (ARDS) is one of the most severe complications of the COVID-19 disease. The role of IL-33 and calpain 1 was previously described in lung infections and lung tissue damage. Our study examined the association between serum calpain 1 activity and IL-33 concentration in patients with COVID-19 ARDS. In the research, we included 80 subjects who had COVID-19 pneumonia and divided them into 2 groups: 40 subjects with ARDS and 40 subjects without ARDS. The basis of the research was the collection of subjects’ data and the sampling of peripheral venous blood. The concentration of IL-33 was determined by the ELISA method and the activity of calpain 1 by the fluorometry method. Our research showed elevated calpain 1 activity and IL-33 concentration in the serum of COVID-19 patients who developed ARDS compared to those who did not develop ARDS and a positive correlation between them was established. Further, a positive correlation was established between the examined parameters and the severity of the disease, proinflammatory markers, and the use of mechanical ventilation. These results indicate a possible association and role of calpain 1 and IL-33 with the development of ARDS in COVID-19 patients.

## 1. Introduction

Coronavirus disease 2019 (COVID-19) is a new disease that appeared in China in 2019 caused by an RNA virus from the coronavirus group, the SARS-CoV-2 virus [1]. From its appearance until today, more than 270 million people have been affected by COVID-19, and more than 6 million people have died [2]. Although in most cases COVID-19 is a mild or moderate disease (especially after the advent of vaccines), severe forms such as acute respiratory distress syndrome (ARDS) occur in some cases [3]. ARDS is an acute respiratory disease characterized by bilateral chest X-ray opacities with severe hypoxemia due to noncardiogenic pulmonary edema [4]. In contrast to the Berlin definition, which defines the temporal occurrence of ARDS within 7 days of disease onset, the ARDS we see at COVID-19 appears on average between the 8th and 12th day of the disease [5]. Available data in the literature show the incidence of ARDS in COVID-19 patients in a wide range from 5% to 35% [6,7,8]. The occurrence of ARDS in COVID-19 patients is associated with an increased mortality rate, ranging from 39 to 79% according to various studies [9]. Although the pathophysiological mechanisms linking COVID-19 and the development of ARDS have not been fully explored and known, the basic fact is that it is associated with an excessive inflammatory response, i.e., the development of a cytokine storm [10,11]. Studies of ARDS in animal models showed increased levels of interleukin 33 (IL-33) in serum and bronchoalveolar lavage of animals in which the development of ARDS was induced [12,13]. Elevated levels of IL-33 have also been found in the serum of patients with ARDS compared with healthy controls [14] and described as a potential predictor of the development of ARDS and poor clinical outcomes [15]. IL-33 is a nuclear factor released by various cells, including alveolar endothelial and epithelial cells, in response to the action of a deleterious factor (so-called alarmin) [16]. IL-33 achieves its proinflammatory effect by binding to transmembrane receptors ST-2 (ST2L) located on the membranes of various inflammatory cells. As a result of this binding, intracellular mechanisms are activated, leading to increased production of inflammatory cytokines, inducing a state of hyperinflammation and a cytokine storm [17,18]. In this context, several papers described IL-33 as an important pathophysiological factor in the development of severe forms of COVID-19 [19,20,21,22,23]. Calcium-dependent proteases from the calpain group, particularly calpain 1, play an important role in mediating damage to the lung parenchyma that we find in conditions such as ARDS. Its role in lung tissue injury has been described in various lung diseases such as bacterial pneumonia, chronic obstructive pulmonary disease, and ventilator-induced lung injury. Increased calpain 1 activity has been associated with inflammation, apoptosis, and fibrosis [24,25,26,27]. Calpain 1 is also involved in the proteolytic cleavage of various proteins and the posttranslational modification of proteins, activating them or increasing their activity [28]. Thus, its effect on the activation of IL-33 has been described. Proteolytic cleavage of the inactive precursor form of IL-33 (pro-IL-33) into its active form has been demonstrated in human epithelial cell models [29]. Current knowledge suggests that calpain 1 may play a dual role in the development of ARDS: a direct cytopathic effect and an increase in the inflammatory response through direct activation of IL-33, which contributes to the development of cytokine storm and ARDS. In addition, the role of calpain 1 has also been described in terms of the internalization of the virus into cells, and its inhibitors are placed in the context of potential drugs for the treatment of COVID-19 [30,31].

The primary objective of our research was to investigate the relationship between calpain 1 activity and serum IL-33 concentration in patients with SARS-CoV-2 pneumonia who developed ARDS and to compare them with patients who did not develop ARDS. The secondary objectives were to compare the levels of calpain 1 activity and IL-33 concentration with the severity of the clinical condition and the levels of inflammatory response markers, and to explore the influence of mechanical ventilation on the levels of calpain 1 activity and IL-33 concentration.

## 2. Materials and Methods

In this case–control study, a total of 80 subjects over the age of 18 years with a diagnosis of pneumonia caused by the SARS-CoV-2 virus were enrolled based on a positive PCR test and radiological findings. Subjects were divided into two groups: the examined and the control group. The examined group (40 subjects) consisted of patients with developed ARDS who required oxygen therapy or mechanical ventilation. The control group (40 subjects) consisted of patients who had not developed ARDS and did not require oxygen therapy or mechanical ventilation. The diagnosis of ARDS was based on the 2012 Berlin definition criteria: (1) the onset of respiratory symptoms within 7 days with gradual progression; (2) the presence of bilateral diffuse radiographic infiltrates which are not associated with atelectasis, pulmonary nodules, or effusions; and (3) the patient’s clinical condition is not associated with heart failure or volume overload. Considering the natural course of SARS-CoV-2 virus infection, in which ARDS may develop during the second and third week of the disease, patients who developed ARDS after the seventh day were also included. Individuals older than 80 years, patients with malignant disease, patients on immunosuppressive therapy, and patients with an existing bacterial nosocomial infection (elevated procalcitonin levels and/or positive microbiological cultures) were not included. The investigation was conducted in the Osijek Clinical Hospital Center during the second and third epidemic waves of COVID-19 disease in the Republic of Croatia. The study was approved by the Ethics Committee of the Faculty of Medicine Osijek, Josip Juraj Strossmayer University of Osijek (number: R2-208/2021). 

The basis of the study was the collection of data on the subject and the collection of peripheral venous blood for the determination of laboratory parameters. Data on the subjects included demographic data (gender, age), the presence of chronic comorbidities, and data on recent COVID-19 disease (duration of the disease, diagnostic workup, severity of the disease, treatment method, and outcome). Disease severity was determined using the MEWS score for all subjects and the APCHE IV score for subjects who developed ARDS. The MEWS scoring system is based on the assessment of vital signs (respiratory rate, heart rate, systolic blood pressure, body temperature, state of consciousness), and the sum of scores ≤2 indicates mild and moderate severity, 3–4 indicates severe, and ≥5 indicates a critical form of the disease [32]. The APCHE IV scoring system is recognized as an important scoring system for determining the severity and risk of death in critically diseased COVID-19 patients [33].

Blood samples were collected between the 10th and 20th day of the disease. The first day is defined as the day on which symptoms of COVID-19 infection initially appeared. The concentrations of inflammatory markers (C-reactive protein [CRP], ferritin, fibrinogen, interleukin-6 [IL-6], and procalcitonin [PCT]), IL-33 serum level, and calpain 1 activity were determined. 

Blood samples for inflammatory biomarkers (CRP, ferritin, IL-6, PCT), calpain 1, and IL-33 were collected in two 4 mL serum-separating tubes (BD Vacutainer, Becton, Dickinson and Company, Franklin Lakes, NJ, USA, SAD). Blood for fibrinogen concentration determination was collected in one tube with sodium citrate (BD Vacutainer, Becton, Dickinson and Company, Franklin Lakes, NJ, USA, SAD). Samples were centrifuged at 1370× *g* for 10 min. CRP, PCT, IL-6, ferritin, and fibrinogen were determined immediately after blood sample collection. For determination of calpain 1 activity, and IL-33 concentration, serum aliquots were frozen at −70 °C until analysis.

CRP and ferritin were measured on biochemistry analyzer Olympus AU680 (Beckman Coulter, Brea, CA, USA) using the latex-enhanced immunoturbidimetric assay. Fibrinogen level was measured using a BCS XP coagulometer (Siemens Healthineers AG, Erlangen, Germany). PCT and IL-6 levels were measured using an electrochemiluminescence immunoassay (ECLIA) on an immunochemistry analyzer, Cobas e411 (Roche Diagnostics GmbH, Mannheim, Germany).

The commercially available ELISA tests (Enzyme-Linked Immunosorbent Assay) were used, following the instructions of the manufacturer, to measure the concentrations of IL-33 (BioVendor—Laboratorni medicina a.s.; Brno, Czech Republic). ELISA tests were performed on the ELISA processor EtiMax 3000 (DiaSorin, Saluggia, Italy).

Calpain 1 activity was determined using a fluorimetric method (Abcam Inc., Cambridge, UK) on Cary Eclipse fluorescence spectrophotometer (Varian Medical Systems, Budapest, Hungary). The fluorometric assay is based on the detection of cleavage of calpain 1 substrate Ac-LLY-AFC that emits blue light (λmax = 400 nm). Upon cleavage of the substrate by calpain 1, free AFC emits yellow-green fluorescence (λmax = 505 nm). The fluorimetric signal of AFC was measured on a fluorescence plate reader. The activity was expressed as a change of Relative Fluorescent Units (RFU) per microliter in one hour.

Based on previous studies focusing on IL-33 and calpain 1, sample size was estimated a priori using G-power 3.1. Power analyses revealed that 80 participants would give 85% power at alpha = 0.05 to detect medium effect size using F tests. Skewness values were determined to be outside normality at the *p* < 0.05 significance level if the value was greater than the absolute value of 3; further, kurtosis values were outside of normality when the absolute value reached 8 or 10. Therefore, the skewness and kurtosis of the variables in this study were within the ±2 absolute value range, indicating that they did not deviate from the assumption of normal distribution. Given this, groups were compared using either *t*-test (e.g., gender differences) or ANOVA (e.g., groups with different severity of COVID-19), whereas relationships between variables of interest were examined using Pearson correlation coefficients. Results are expressed as mean and standard deviation. Statistical significance was set at *p* < 0.05, and for correlation analysis *p* < 0.01. Statistical analysis was carried out using the MedCalc Statistical software version 12.4.0.0. (MedCalc Software, Mariakerke, Belgium).

## 3. Results

In total, 80 subjects participated in this study, equally represented in the examined (*n* = 40) and control (*n* = 40) groups. A total of 60% (*n* = 48) were men. The mean age of all subjects was 60.47 ± 10.79 years, which did not differ significantly with regard to gender, t(78) = 0.870, *p* = 0.387. The baseline characteristics of subjects are shown in Table 1.

In both groups of subjects, the magnitude and direction of the correlation between calpain1 activity and IL-33 concentration were almost identical. The correlation between the observed parameters in subjects with ARDS was r = 0.95 (*p* = 0.001). Serum calpain 1 activity and IL-33 concentration were statistically significantly higher in subjects who developed ARDS than in those who did not. In ARDS, the effect sizes for these differences were large (Table 2). Considering the statistically significant age difference between these two studied groups, t(38) = 3.757, *p* = 0.001, it was taken as a covariate, whereas in subjects without ARDS it was r = 0.97 (*p* = 0.001). As calpain 1 activity increased, so did the concentration of IL-33.

Considering the severity of ARDS, indicated by the ratio between the partial pressure of oxygen in arterial blood and the fraction of inspired oxygen (PaO_2_/FiO_2_) [4], mild ARDS (PaO_2_/FiO_2_ > 200) was present in 2 subjects (5%), 21 (52.5%) of them had moderate ARDS (PaO_2_/FiO_2_ 100–200), and 17 of them (42.5%) had severe ARDS (PaO_2_/FiO_2_ ≤ 100). The highest levels of calpain 1 activity and IL-33 concentration were found in subjects with severe ARDS and the lowest in those with moderate and mild ARDS (Table 3).

Regarding the severity of the disease, which was evaluated according to the MEWS scoring system, 40% of the subjects had a mild and moderate form of the disease, 10% had a severe form, and 50% of the subjects had a critical form. Since these three groups also differed from each other in terms of age, F(2.77) = 12.88, *p* = 0.001, the analysis was performed with the age control of the subjects. The highest values of serum calpain 1 activity and IL-33 concentration were found in subjects with a critical form of the disease, while they did not differ significantly between subjects with a severe and a mild and moderate form of the disease (Table 4). 

In all three groups of subjects, the correlations between the levels of calpain 1 activity and IL-33 concentration were similar with respect to the severity of the clinical presentation. In subjects with mild, moderate, and severe forms, it was r = 0.97, *p* = 0.001, while in those with critical form, it was r = 0.96, *p* = 0.001. Higher calpain 1 activity levels were associated with higher concentrations of IL-33 in all three groups.

The APACHE IV score was calculated only for subjects with ARDS and was 75.85 ± 10.67. In determining the association between the value of the APACHE IV score and the observed parameters, a statistically significant, positive, and moderate association was found with the activity of calpain 1 (r = 0.50, *p* = 0.001) and the concentration of IL-33 (r = 0.54, *p* = 0.001).

With regard to the levels of the inflammatory response markers, statistically significantly higher levels of CRP, ferritin, fibrinogen, and IL-6 and lower levels of the ratio IL-33/IL-6 and IL33/CRP were found in subjects who developed ARDS compared to those who did not. The effect sizes for these differences were large (Table 5). 

Table 6 shows the associations between serum calpain 1 activity and IL-33 concentration with inflammatory response markers in all subjects and within all tested groups. Overall, calpain 1 activity values and IL-33 concentrations were low to highly significantly associated with most markers of the inflammatory response. The analysis was carried out considering the age of the subjects.

A comparison of serum calpain 1 activity and IL-33 concentration with respect to the degree of respiratory support is shown in Table 7. Patients in the control group did not require oxygen, whereas patients in the examined group required some form of ventilatory support. Sixteen subjects received classical oxygen therapy, and twenty-four subjects required mechanical ventilation. These groups differed from each other in terms of age, F(2,77) = 7.51, *p* = 0.001; therefore, the analysis was performed controlling for the age of the subjects. Subjects who were mechanically ventilated had significantly higher levels of calpain 1 and IL-33 concentration compared to other subjects. Subjects who did not require oxygen had the lowest values. 

In subjects receiving mechanical ventilation, correlations between serum calpain 1 and IL-33 concentrations and baseline settings of mechanical ventilation were also examined. Only a statistically significant correlation was found between FiO_2_ value and serum calpain 1 activity (r = 0.41, *p* = 0.05) and IL-33 concentration (r = 0.42, *p* = 0.04). We did not find a correlation between positive end-expiratory pressure (PEEP) and inspiratory pressure support (PS). In addition, a statistically significant correlation was found between the observed parameters and peak inspiratory pressure (PIP) and driving pressure (ΔP). Mechanically ventilated subjects who achieved higher PIP had higher values and activities of calpain 1 activity (r = 0.66, *p* = 0.001) and IL-33 (r = 0.69, *p* = 0.001). Similar results were obtained for ΔP: calpain 1 activity (r = 0.67, *p* = 0.001), and IL-33 (r = 0.64, *p* = 0.001). Table 8 shows the average values of the tested parameters with regard to the ventilation modality. The comparison of arithmetic means showed that the lowest values of calpain 1 activity and IL-33 were documented in subjects ventilated in the CPAP + PS modality; nevertheless, there were only two subjects.

## 4. Discussion

Our study showed increased calpain 1 activity and IL-33 concentration in the serum of COVID-19 patients who developed ARDS compared with those who did not, with a positive correlation between these two values. In addition, a positive correlation was found between the tested parameters and the severity of the disease, inflammatory markers and the use of mechanical ventilation.

Elevated levels of IL-33 in serum have also been found in other patients with ARDS. In their work, Halat et al. found that patients with polytrauma who initially had higher serum IL-33 levels were more likely to develop ARDS and associated this with worse clinical outcomes, i.e., they described it as a predictor of mortality [15]. 

These results are consistent with animal model studies indicating a role for IL-33 in the development of ARDS. In the work of Liang et al., who used lipopolysaccharide to induce acute lung injury in rats, elevated levels of IL-33 were detected in serum and bronchoalveolar lavage in all rats that developed severe lung injury. Tracheal administration of neutralizing anti IL-33 antibodies reduced edema and inflammatory infiltration of the lung parenchyma [34]. Moreover, increased expression of IL-33 was observed in wild-type mice in which ARDS was induced with lipopolysaccharide, whereas this was not the case in IL-33 knockout mice, which showed less pronounced inflammation and damage to the lung parenchyma [35]. The above-mentioned studies support the role of IL-33 as an alarmin that is released in conditions of damage to alveolar epithelial and endothelial cells and via transmembrane ST receptors expressed on inflammatory cells stimulates increased production of pro-inflammatory cytokines and supports and enhances the hyperinflammatory response. This assumption was described by Furci et al. in their review paper, in which they present IL-33 as an essential driver of the hyperinflammatory response in COVID-19 infection and describe it as a potential therapeutic target in the prevention of severe clinical forms [22]. The association of IL-33 expression with increased production of inflammatory cytokines (IL-1β, IL-6, TNF-α, IL-12, and IL-23) in the serum of COVID-19 patients was demonstrated by Marković et al. in their work in which IL-33 was investigated as a possible predictor of the severity of the COVID-19 disease [36]. On the contrary, Gaurav et al. obtained results in their research in which they studied the postmortem expression of IL-33 in the lung tissue of COVID-19 patients and a control group consisting of patients with chronic lung diseases [21]. Significantly lower expression of IL-33 was found in the lung tissue of COVID-19 patients compared with the control group [21], which may be related to the possibility of depletion of IL-33 as well as its degradation, which cannot be detected by commercially available antibodies.

Elevated values of calpain 1 activity in our study of COVID-19 patients with developed ARDS are consistent with earlier studies that indicate the role of calpain 1 in acute lung parenchymal damage. In their research, Du et al. demonstrated in their study that the administration of calpain inhibitors reduced acute burn-induced lung injury in rats. Rats that received a calpain inhibitor (MDL28170) immediately before burn exposure had reduced calpain 1 expression and milder lung parenchymal injury [24]. In their study, Yi et al. found elevated values of serum calpain activity in patients who developed acute lung damage after cardiac surgery and proposed it as a potential biomarker of acute lung damage after cardiopulmonary bypass [37]. The role of calpain 1 in lung parenchymal damage has also been described in other diseases such as pneumonia, chronic obstructive pulmonary disease, asthma, and lung damage associated with mechanical ventilation [25,27,38,39,40]. The role of calpain 1 in enhancing the inflammatory response, cell apoptosis, and fibroproliferative processes supports its increased levels in patients with COVID-19 ARDS, as demonstrated in our study. Several publications have identified the calpain system as a potential therapeutic target for the treatment and prevention of the development of more severe forms of COVID-19, i.e., ARDS [30,31].

Hayakawa et al. pointed out the correlation between calpain 1 and IL-33 by describing the post-translational cleavage of inactive pro-IL-33 into the active form of IL-33 mediated by calpain 1 in human epithelial cell cultures [29]. Scott and colleagues published a similar report demonstrating this proteolytic cleavage in a mouse lung model exposed to the extract of the fungal allergen Alternaria alternata [41]. The results of our study are consistent with these conclusions, as our subjects who had higher levels of calpain 1 activity also had higher levels of IL-33. This was particularly pronounced in subjects who developed ARDS, although this association was also found in subjects who did not develop ARDS. Furthermore, we see a link between these two factors in the fact that IL-33, as an important factor in stimulating the immune response, enhances chemotaxis of neutrophils into the lung parenchyma, and neutrophils are one of the sources of calpain 1 [19,42].

In our study, the levels of serum calpain 1 activity and IL-33 concentration correlated positively with the severity of ARDS. As expected, we found the highest values in subjects with severe ARDS, whereas those with moderate and mild ARDS had lower values.

Comparing the values of calpain 1 activity and IL-33 concentration with the severity of COVID-19 disease in all subjects, the highest values of both parameters were obtained in critically ill subjects (MEWS ≥ 5), whereas we found no significant difference in the values between severely ill subjects (MEWS 3–4) and moderately and moderately ill patients (MEWS ≤ 2). These results were expected because all subjects in the severely ill group had developed ARDS, for which we demonstrated statistically significant higher values of both parameters compared with subjects without ARDS. A positive correlation between IL-33 and the severity of COVID-19 disease was also found by other authors [36,43,44], while the role of calpain 1 in this regard has not yet been investigated. Moreover, a positive correlation was found between calpain 1 and IL-33 and the value of the APACHE IV score, which is also to be expected, since subjects with a severe form of ARDS had a higher APACHE IV score.

Markers of inflammatory response (CRP, ferritin, fibrinogen, IL-6) were statistically significantly higher in subjects who developed ARDS in our study, which is consistent with the fact that hyperinflammatory state and cytokine storm play an important role in the pathophysiology of ARDS [45]. In addition, we found a positive correlation between the levels of calpain 1 activity and IL-33 with almost all markers of inflammatory response, supporting the theory of their role in the development of a cytokine storm [22,46]. In addition to the previously described proteolytic cleavage of pro-IL-33, calpain 1 directly stimulates the release of other inflammatory cytokines such as IL-6, IL-12, and IL-17, thus supporting the condition of hyperinflammation [47].

When analyzing the effect of ventilatory support in our subjects on the values of the studied parameters, we obtained the expected higher values in the subjects who received mechanical ventilation compared with those who received classical oxygen therapy and those who did not require oxygen. This is consistent with the fact that the mechanically ventilated subjects belonged to the group of subjects with a severe form of ARDS, in whom the highest values of calpain 1 and IL-33 activity were measured. However, mechanical ventilation itself may have contributed to these elevated levels. In animal models, Liu et al. and Ding et al. have shown that mechanical ventilation is associated with increased secretion of IL-33 and lung injury [48,49]. Ding explained this statement with the occurrence of mechanical stress as an inducer of IL-33 that is produced in the alveoli during mechanical ventilation depending on tidal volume [48]. In our study, mechanically ventilated subjects who achieved higher PIP and ΔP during ventilation had higher values of the observed parameters, supporting this conclusion because they had greater mechanical stress from pressure loading. In addition, we found higher values of the parameters studied in subjects ventilated with a higher FiO_2_, suggesting that oxidative stress also increases the expression of IL-33 [50,51]. Mechanical ventilation is also associated with increased calpain activation. Liu et al. showed that mechanical ventilation with high tidal volume in mice causes rapid and permanent activation of calpain, which is accompanied by the development of inflammation, increased production of TNF-α and IL-6, increased permeability of blood vessels, and the occurrence of pulmonary edema [27]. We did not find any influence of the mechanical ventilation modality on the values of the examined parameters.

## 5. Conclusions

Our study showed that subjects with COVID-19 pneumonia who developed ARDS had significantly higher values of calpain 1 activity and IL-33 serum levels compared to those who did not develop ARDS. Moreover, these values were higher in patients with a severe form of ARDS, compared to those who had a moderate or mild form. Also, our study suggests upstream regulatory relationship between clapain 1 and IL-33. Further, we found a positive correlation between the examined parameters and the severity of the disease, markers of inflammatory response, and the use of mechanical ventilation. These results suggest a possible association and role of calpain 1 and IL-33 with the development of ARDS in COVID-19 patients. Further research with a larger number of subjects is needed to analyze this mechanism of action and to determine its possible therapeutic potential.

## Figures and Tables

**Table 1:** Baseline characteristics of the subjects.

	All Subjects (*n* = 80)	Control Group (*n* = 40)	Examined Group (*n* = 40)	*p*-Value
Age (*M* ± *SD*)	60.47 ± 10.79	56.28 ± 11.42	64.68 ± 8.34	0.001
Gender				
Men	48 (60.0%)	23 (57.5%)	25 (62.5%)	0.648
Women	32 (40.0%)	17 (42.5%)	15 (37.5%)	
Comorbidities				
Without comorbidities	14 (17.5%)	11 (27.5%)	3 (7.5%)	0.019
With comorbidities	66 (82.5%)	29 (72.5%)	37 (92.5%)	
Disease severity				
MEWS * 2	32 (40.0%)	32 (80.0%)	0 (0.0%)	0.001
MEWS * 3	8 (10.0%)	8 (20.0%)	0 (0.0%)	
MEWS * 4	0 (0.0%)	0 (0.0%)	0 (0.0%)	
MEWS * 5	40 (50.0%)	0 (0.0%)	40 (100%)	
Outcome				
Survived	52 (65.0%)	40 (100%)	12 (30.0%)	0.001
Died	28 (35.0%)	0 (0.0%)	28 (70.0%)	

* Modified Early Warning Score; *M* = mean; *SD* = standard deviation.

**Table 2:** Comparison of serum calpain 1 activity and IL-33 concentration with regard to the occurrence of ARDS.

	All Subjects (*n* = 80)		Control Group (*n* = 40)		Examined Group (*n* = 40)		ANCOVA	Simple Size
	*M*	*SD*	*M*	*SD*	*M*	*SD*	*F-Ratio*	η_p_^2^
Calpain 1 activity (RFU/µL)	2.72	2.41	0.99	0.35	4.46	2.33	66.24 ***	0.462
IL-33 concentration (µg/L)	5.28	1.49	4.12	0.55	6.45	1.18	95.83 ***	0.554

*** *p* < 0.001; η_p_^2^ = partial eta squared (0.001 = small effect size; 0.06 = medium effect size; 0.14 = large effect size).

**Table 3:** Comparison of serum calpain 1 activity and IL-33 concentration with regard to the severity of ARDS.

	Mild ARDS (*n* = 2)		Moderate ARDS (*n* = 21)		Severe ARDS (*n* = 16)		ANOVA	Post Hoc Test
	*M*	*SD*	*M*	*SD*	*M*	*SD*	*F-Ratio*	*Bonferroni*
Calpain 1 activity (RFU/µL)	2.39	0.41	3.23	1.02	6.09	2.44	12.45 ***	Severe > Mild * Severe > Moderate ***
IL-33 concentration (µg/L)	5.21	0.15	5.81	0.64	7.35	1.12	14.86 ***	Severe > Mild ** Severe > Moderate ***

*** *p* < 0.001; ** *p* < 0.01; * *p* < 0.05; *M* = mean; *SD* = standard deviation; ANOVA = Analysis of variance (continuous variables).

**Table 4:** Comparison of serum calpain 1 activity and IL-33 concentration with regard to disease severity (MEWS scoring system).

	Mild/Moderate Disease (*n* = 32)		Severe Disease (*n* = 8)		Critical Disease (*n* = 40)		ANOVA	Post Hoc Test
	*M*	*SD*	*M*	*SD*	*M*	*SD*	*F-Ratio*	*Bonferroni*
Calpain 1 activity (RFU/µL)	0.88	0.31	1.41	0.05	4.47	2.33	43.65 ***	Critical > Mild *** Critical > Severe ***
IL-33 concentration (µg/L)	3.96	0.50	4.75	0.08	6.45	1.18	70.12 ***	Critical > Mild *** Critical > Severe ***

*** *p* < 0.001; *M* = mean; *SD* = standard deviation; ANOVA = Analysis of variance (continuous variables).

**Table 5:** Comparison of the inflammatory response markers regard to the incidence of ARDS.

	All Subjects (*n* = 80)		Control Group (*n* = 40)		Examined Group (*n* = 40)		ANCOVA	Simple Size
	*M*	*SD*	*M*	*SD*	*M*	*SD*	*F-Ratio*	η_p_^2^
CRP (mg/L)	120.12	71.33	70.34	20.77	169.90	69.24	60.13 ***	0.439
Ferritin (µg/L)	692.65	358.08	444.24	148.18	941.05	333.47	67.18 ***	0.466
Fibrinogen (g/L)	5.62	2.41	3.64	1.51	7.60	1.20	128.89 ***	0.626
IL-6 (ng/L)	279.60	237.14	124.17	44.29	435.04	249.79	45.25 ***	0.370
IL-33/IL-6	28.45	15.99	37.01	13.26	19.89	13.84	18.46 ***	0.193
IL-33/CRP	0.51	0.18	0.62	0.17	0.41	0.10	30.62 ***	0.285

*** *p* < 0.001; η_p_^2^ = partial eta squared (0.001 = small effect size; 0.06 = medium effect size; 0.14 = large effect size).

**Table 6:** Pearson correlation of serum calpain 1 activity and IL-33 concentration with inflammatory response markers.

	All Subjects (N = 80)		Control Group (*n* = 40)		Examined Group (*n* = 40)	
	Calpain 1 Activity (RFU/µL)	IL-33 Concentration (µg/L)	Calpain 1 Activity (RFU/µL)	IL-33 Concentration (µg/L)	Calpain 1 Activity (RFU/µL)	IL-33 Concentration (µg/L)
IL-33 (µg/L)	0.97 ***	─	0.95 ***	─	0.97 ***	─
CRP (mg/L)	0.78 ***	0.80 ***	0.39 ***	0.43 ***	0.61 ***	0.64 ***
Ferritin (µg/L)	0.63 ***	0.64 ***	0.21	0.20	0.33 *	0.27
Fibrinogen (g/L)	0.67 ***	0.74 ***	0.57 ***	0.59 ***	0.38 *	0.36 *
IL-6 (ng/L)	0.69 ***	0.69 ***	0.72 ***	0.67 ***	0.47 ***	0.44 ***
IL-33/IL-6	−0.29 **	−0.36 **	−0.49 ***	−0.40 **	0.08	0.06
IL-33/CRP	−0.45 ***	−0.49 ***	−0.06	−0.09	−0.25	−0.26

*** *p* < 0.001; ** *p* < 0.01; * *p* < 0.05.

**Table 7:** Comparison of serum calpain 1 activity and IL-33 concentration with regard to the level of respiratory support.

	Without the Use of Oxygen (*n* = 40)		Classic Oxygen Therapy (*n* = 16)		Mechanical Ventilation (*n* = 24)		ANCOVA	Simple Size
	*M*	*SD*	*M*	*SD*	*M*	*SD*	*F-Ratio*	η_p_^2^
Calpain 1 activity (RFU/µL)	0.99	0.35	2.60	0.51	5.71	2.24	84.18 ***	0.69
IL-33 concentration (µg/L)	4.12	0.55	5.40	0.27	7.16	1.02	117.16 ***	0.76

*** *p* < 0.001; *M* = mean; *SD* = standard deviation; η_p_^2^ = partial eta squared (0.001 = small effect size; 0.06 = medium effect size; 0.14 = large effect size).

**Table 8:** Average values of serum calpain 1 activity and IL-33 concentration with regard to the modality of mechanical ventilation.

Ventilation Modality *	Number of Subjects (*n*)	Calpain 1 Activity (RFU/µL)		IL-33 Concentration (µg/L)	
		*M*	*SD*	*M*	*SD*
CPAP + PS	2	1.95	0.07	5.10	0.00
BiPAP	6	5.92	2.26	7.23	0.74
SIMV	13	5.82	2.42	7.24	1.23
MMV	3	5.70	2.69	7.02	0.89

* CPAP + PS—Continuous Positive Airway Pressure + Pressure Support; BiPAP—Bilevel Positive Airway Pressure; SIMV—Synchronized Intermittent Mandatory Ventilation; MMV—Mandatory Minute Ventilation; *M* = mean; *SD* = standard deviation.

## Data Availability

The datasets generated and/or analyzed during the current study are not publicly available due to the fact that individual privacy could be compromised; however, they are available from the corresponding author on reasonable request.
